# Psychedelics and Consciousness: Distinctions, Demarcations, and Opportunities

**DOI:** 10.1093/ijnp/pyab026

**Published:** 2021-05-14

**Authors:** David B Yaden, Matthew W Johnson, Roland R Griffiths, Manoj K Doss, Albert Garcia-Romeu, Sandeep Nayak, Natalie Gukasyan, Brian N Mathur, Frederick S Barrett

**Affiliations:** 1 Department of Psychiatry and Behavioral Sciences; 2 Center for Psychedelic and Consciousness Research; 3 Department of Neuroscience; 4 Johns Hopkins University School of Medicine, Baltimore, Maryland, USA; 5 Department of Pharmacology, University of Maryland School of Medicine, Baltimore, Maryland, USA

**Keywords:** Altered states of consciousness, consciousness, LSD, psilocybin, psychedelics

## Abstract

Psychedelic substances produce unusual and compelling changes in conscious experience that have prompted some to propose that psychedelics may provide unique insights explaining the nature of consciousness. At present, psychedelics, like other current scientific tools and methods, seem unlikely to provide information relevant to the so-called “hard problem of consciousness,” which involves explaining how first-person experience can emerge. However, psychedelics bear on multiple “easy problems of consciousness,” which involve relations between subjectivity, brain function, and behavior. In this review, we discuss common meanings of the term “consciousness” when used with regard to psychedelics and consider some models of the effects of psychedelics on the brain that have also been associated with explanatory claims about consciousness. We conclude by calling for epistemic humility regarding the potential for psychedelic research to aid in explaining the hard problem of consciousness while pointing to ways in which psychedelics may advance the study of many specific aspects of consciousness.

The resurgence of psychedelic research has provided tools for researchers who study mental processes such as perception, affect, and cognition (Johnson et al., 2019). Beyond these routine subjects of scientific inquiry for the brain and cognitive sciences, some have expressed hopes that psychedelics may somehow help to explain consciousness (e.g., the sub-title of Michael Pollan’s bestselling book on psychedelics begins, “What the New Science of Psychedelics Teaches Us About Consciousness…” (Pollan, 2019). Even the term “psychedelic” itself refers to consciousness, as it comes from the Greek for “mind”—psyche (ψυχή)—and “manifesting”—delic (δηλείν) (Osmond, 1957). By consciousness here, in the broadest possible sense before introducing further distinctions, we mean to refer to the mental state of basic subjective awareness or first-person experience (or the “what it is like” to be a system or organism) that, for example, occurs after one awakens from the lack of consciousness of deep sleep or anesthesia (e.g., Nagel, 1974; Chalmers, 1995, 1996; Metzinger, 2000; Seth et al., 2006; Blackmore, 2013; Koch et al., 2016).

Psychedelic substances produce unusual and compelling changes in conscious experience that have prompted some to propose that psychedelics may provide unique insights into the nature of consciousness. While psychedelics can and are being used to study their effects on consciousness (or more precisely, on the contents of consciousness), the idea that psychedelics can and are being used to explain the hard problem of consciousness (defined below) is quite another matter (e.g., Blackmore, 2013; Letheby, 2015; Bayne and Carter, 2018; Johnson, 2020). Here, we argue that the relationship between psychedelics and consciousness hinges on what is meant by the term “consciousness,” which researchers tend to use in different ways at different times (e.g., Chalmers, 1995; Velmans, 2009). As Johnson (2020) argues, more care is required regarding the use of the term consciousness, particularly among those who study psychedelics. Here we describe issues at the intersection between psychedelics and different senses of the term consciousness and conclude by calling for high levels of epistemic humility around the potential for psychedelics to aid in explaining consciousness, while pointing to some specific senses of the term consciousness that psychedelic research may help to illuminate.

## The hard and easy Problems of Consciousness

The philosophical concepts of the “hard problem” and the “easy problem(s)” of consciousness are among the most basic distinctions––a debate with historical roots that remains a lively contemporary discourse (Chalmers, 1995). The hard problem of consciousness refers to explaining what phenomenal consciousness is and how it comes to be. Phenomenal consciousness can be defined as the first person subjective “what it is like” to be an organism (Nagel, 1974); these subjective sensations are also referred to as “qualia” (Lewis, 1929). In this sense, the hard problem is concerned with explaining what the immediate, subjective experience of being (i.e., phenomenal consciousness) is and how it relates to objectively observable phenomena such as brain activity and behavior. The hard problem of consciousness is currently not scientifically answered, and it is not clear that a scientific answer is even possible, which is why it is called “a hard problem.” For this reason, the hard problem is often described in terms of the “explanatory gap” (Levine, 1983). This phrase may be an understatement––there is far more than a gap, but rather a yawning chasm between our current scientific understanding and the prospect of explaining the hard problem of consciousness. At present, there is little reason to think that psychedelics will bring us any closer to closing the explanatory gap.

In contrast, the so-called “easy problems” of consciousness consist of a variety of distinct problems that are plausibly explainable (e.g., how attention, perception, and the deliberate control of behavior work). One common example of an easy problem (which in actual fact is quite a difficult scientific subject) is explaining how light on the retina is eventually perceived in the visual field. Even if they are not currently explained, it seems clear that these and related questions can possibly be explained to some degree or in some mechanistic fashion through current scientific methods given sufficient time and effort. Understanding these mental processes involves describing the contents of consciousness. In contrast to the hard problem, psychedelic research may be brought to bear on some of the “easy problems.”

One of the many reasons that the hard problem of consciousness is difficult (or maybe impossible) to scientifically address is that phenomenal consciousness cannot be observed directly by anyone other than a given conscious entity. This is referred to as “the problem of other minds” (Blackburn, 1994). To illustrate, the reader knows that she or he is conscious, but cannot be certain that anyone else is conscious. Strictly speaking, the best one can do is infer consciousness in others on the basis that others seem to be similar kinds of beings as oneself. This poses serious problems for the scientific method regarding consciousness. Even if it were possible to engineer the creation of consciousness (e.g., with artificial intelligence), it is unclear how one could prove whether consciousness had indeed been created. One could ask another entity if it were conscious (e.g., an artificial intelligence), but there would be no way to verify its self-report. Block (1995) distinguished between “access consciousness,” which involves the availability of information and ability to use it in reasoning processes, and “phenomenal consciousness” (introduced in the previous paragraph), which involves the feeling quality of being aware. One could establish, for example, whether an artificial intelligence has access consciousness (e.g. via some form of the Turing test; Turing, 1950), but could not establish whether an artificial intelligence has phenomenal consciousness. The identical problem of verifiability presents itself when attempting to assess consciousness in another person. Some of these issues extend to the study of the psychological processes that consist of the contents of consciousness and, by extension, the various easy problems of consciousness, at least insofar as subjective access to the content of one’s consciousness remains private. However, a difference is that the contents of consciousness (thoughts, emotions, perceptions, etc.) can be caused and manipulated to some extent within other people, and these changes can be more directly reported and measured, whereas phenomenal consciousness itself cannot be caused or so clearly operationally observed in this manner (and its causation cannot be verified).

While there is no current scientific explanation of phenomenal consciousness, there are a number of philosophical theories about its nature. These theories can be grouped into 3 broad categories: materialist, dualistic, and monistic (e.g., Chalmers, 2003). Materialist theories, favored by many scientists, regard phenomenal consciousness as identical to brain states. However, materialists would need to explain how physical processes (e.g., the brain) give rise to “qualia,” the “what it feels like” quality of being conscious, or how subjective experience arises from interaction of material entities (e.g., atoms, neurons) that presumably do not possess such phenomenal consciousness themselves. Some (e.g., Dennett, 1993) see the problem of qualia (and indeed the whole notion of the hard problem) as a pseudoproblem, arguing that there is nothing to explain, while others see this dismissal as ignoring the fundamental phenomena (e.g., Chalmers, 1996). Dualistic theories consider phenomenal consciousness as distinct from matter (i.e., that phenomenal consciousness is essentially different from matter and brain states, though these may interact in ways yet to be precisely determined). Dualistic theories would need to explain what evidence there is for this perspective (beyond intuitions) as well as how something non-physical (i.e., consciousness) could interact with physical matter (e.g., the brain), and how this can be ascertained (or falsified) empirically. Monistic views such as neutral monism conceive of phenomenal consciousness and matter as part of the same basic stuff, which is neither inherently matter nor mind. This view is often associated with a view called panpsychism, which posits that all matter (not just brains) is in some way conscious. Due to the issues such as those related to verifiability, it is not clear to us that psychedelics can inform challenges posed by each of the philosophical theories about the nature of consciousness––at least at present. Psychedelics may, though, impact beliefs about these different theories of phenomenal consciousness under some circumstances, which we will return to later in this review.

Scientists tend to ignore the hard problem of consciousness and generally adopt methodological materialism to address the easy problems of consciousness. This is the principal domain of research of many scientific disciplines (e.g., psychology, cognitive science, neuroscience, and psychopharmacology). Thus, the term “consciousness” is sometimes used to refer to the wide array of the contents of the mind in general (e.g., perceptions, thoughts, feelings, and emotions). This all-inclusive use of the term consciousness is so broad, referring to all of aspects of the contents of consciousness within the range of scientific inquiry in psychology and neuroscience, that it has limited use.

There are a few scientific theories that attempt to explain how phenomenal consciousness appears unitary (perceptual, cognitive, and affective processes appear seamlessly integrated) in terms of neural correlates, a problem referred to as the neural correlates of consciousness. The global workspace theory of consciousness (Baars, 1993; Dehaene and Naccache, 2001) focuses on how disparate mental processes combine to create the unitary “cinematic” quality of conscious experience. The integrated information theory of consciousness (Tononi, 2004) defines consciousness in terms of the level of complexity of ordered information and attempts to operationalize this with Φ (“phi”; Tononi, 2008). Although it would be interesting to investigate how psychedelic states relate to Φ, it is not clear how this would improve our understanding of the hard problem of consciousness. For example, relatively simple digital logic gates (e.g., XOR gate), which intuitively seem non-conscious, can generate large amounts of Φ (Cerullo, 2015). It is also not clear that the assertion of complexity itself being a measure of consciousness is tenable. Increasing or decreasing Φ or dissecting and understanding the global workspace would not explain how physical processes give rise to qualia, and though testing psychedelics in these paradigms may be interesting, psychedelics do not appear to bear on these deeper issues.

## Neuroscientific Models of Psychedelics and their Impact on Understanding Consciousness

Despite the foregoing issues with addressing the hard problem of consciousness, or explaining phenomenal consciousness, some have claimed that neurological theories or models of psychedelic effects might illuminate the subject of phenomenal consciousness. We briefly review 3 popular theories or models of this kind describing the proposed role of psychedelics and noting claims portending that these theories or models may explain how phenomenal consciousness arises from the brain. While it is unclear how any of these models could provide information relevant to the hard problem, each of them do generate interesting predictions regarding various easy problems of consciousness that are scientifically tractable.

Relaxed beliefs under psychedelics (REBUS; Carhart-Harris and Friston, 2019) was proposed as a “unifying model” of the effects of psychedelics on brain function that attempts to explain their subjective effects. The REBUS model largely relies on the free energy principle (FEP; Friston, 2010), which claims that living systems (technically, any system that possesses a boundary called a Markov blanket) seek to reduce uncertainty and do this largely through predictive processes (Friston, 2010; Carhart-Harris and Friston, 2019). REBUS purportedly integrates FEP with the “entropic brain hypothesis” (Carhart-Harris, 2014), which proposes that levels of entropy (disorder or randomness) in brain function will correlate with the subjective diversity and vividness of a given subjective state. According to the entropic brain hypothesis, psychedelics increase brain entropy and therefore result in more diversity and vividness in subjective awareness. The REBUS theory proposes a specific role of increased entropy and decreased “top-down” control of the default mode network (DMN) as central to psychedelic drug effects on the contents of consciousness, such as perceptual alterations. REBUS is slightly more precise than the entropic brain hypothesis insofar as it specifies the primary locus of entropy that is impacted by psychedelics, which is in the “precision weighting of prior beliefs encoded in the spontaneous activity of neuronal hierarchies,” primarily in the DMN (Carhart-Harris and Friston, 2019, 319). REBUS proposes that a relaxation of top-down priors allows bottom-up information to exert more influence in brain function, learning, and perception. In regard to explaining phenomenal consciousness, it has been claimed that through prediction errors of interoceptive processes, FEP can “explain the “‘how and why’ it feels like something within the system, for the system” (Solms and Friston, 2018, 216). That is, this model seeks to explain the effects of psychedelics, and at least part of it has been postulated as a way to explain phenomenal consciousness.

Support for the entropic brain hypothesis includes increases in some measures of entropy in neuroimaging data during the acute effects of psychedelics (Carhart-Harris, 2018), although we note that these findings must be considered preliminary due to the small sample size of the 2 studies that form the basis for many of these speculations. Furthermore, entropy has been inferred or calculated in many different ways; these include a decrease in synchronization of high-frequency electrical (Riba et al., 2002, 2004; Kometer et al., 2015) or magnetic (Muthukumaraswamy et al., 2013; Carhart-Harris et al., 2016) oscillations recorded at the scalp, Shannon’s entropy in node-wise (Carhart-Harris et al., 2014) or edge-wise (other) variance of low-frequency fluctuations in blood oxygenation level–dependent signal, or entropy of dynamic functional connectivity (Doss et al., 2020). Müller et al., 2021 has reported similar fMRI connectivity changes after administration of a selective serotonin reuptake inhibitor (SSRI), 3,4-Methylenedioxy methamphetamine (MDMA), and classic psychedelics, which raises questions regarding the specificity of these findings to classic psychedelics. Rather than providing broad conceptual replications of increased brain entropy during psychedelic states, these very different measures may instead reflect different sources of signal and noise and, therefore, potentially implicate distinct systems-level neural mechanisms. Thus, it is unclear whether these various measures provide support, or even properly test, the hypothesis that entropy causally mediates the diversity or vividness of any given subjective state.

Some observations appear to count against the REBUS model. For example, if psychedelics decrease prediction capacity, then surprise (or more technically, prediction errors) should increase, but evidence for this has been mixed (Umbricht et al., 2003; Schmidt et al., 2012; Vollenweider and Preller, 2020). The wide, nearly non-specific reporting of changes in entropy across various measures of brain function parallels a lack of specificity in what constitutes “top-down”/higher or “bottom-up”/lower levels of brain functioning. Counter to any account of decreased top-down control of bottom-up information, LSD has been shown to increase information flow from the parahippocampal gyrus (which in this example would be nearly unequivocally higher level) to visual cortex (which in this example would be nearly unequivocally lower level) (Kaelen et al., 2016). The specificity of the DMN in neural effects of psychedelics is also in question, as nearly all reports of DMN changes under psychedelics also report equal or greater changes in task-positive functional brain networks (e.g., Carhart-Harris et al., 2014; Roseman et al., 2014; Mason et al., 2020). REBUS seems to provide a hypothesis (e.g., entropic brain; Carhart-Harris, 2018) and some grounding to concepts expressed in the free energy principle (Friston, 2010). One proposed implication of the REBUS model is that psychedelics might be efficacious in the treatment of disorders of consciousness, that is, vegetative and minimally conscious states secondary to acquired brain injury (Scott and Carhart-Harris, 2019). Scott and Carhart Harris (2019) highlight that brain complexity (e.g., measured by Lempel-Ziv compressibility) is positively correlated with states of awareness and they suggest that psychedelic-induced increases in brain complexity may restore or improve awareness in patients with disorders of consciousness (to date, no studies on the effects of psychedelics in this population have been completed). It also appears possible that REBUS may help to explain changes to one’s sense of self-awareness, but self-awareness and related changes in subjectivity remain well within the bounds of the easy problem of consciousness. REBUS thus generates interesting hypotheses to test but little to no hope for gaining traction on the hard problem.

The cortico–striato–thalamo–cortical (CSTC) model addresses well-established circuits between the cortex and the thalamus that are necessary for control of sensory information flow to the cortex and by extension the regulation of awareness and attention (Vollenweider and Geyer, 2001). This model emphasizes the impacts of psychedelics and specifically 5-HT_2A_ receptor activation on cortico-thalamic circuits to explain the subjective effects of psychedelics (Geyer and Vollenweider, 2008). Specifically, the model proposes that psychedelics impede sensory gating functions of the thalamus, allowing increased sensory and interoceptive information flow from thalamus to cortical regions. This reduction in sensory gating is proposed to lead to a literal kind of sensory overload of the cortex that results in both perceptual effects and cognitive changes that are observed during the acute effects of psychedelics. Proponents of this model also refer to theories of consciousness that prioritize these same cortico-thalamic circuits, suggesting that activity in these loops contributes to conscious experience. Preller and colleagues have suggested that “[the thalamus] also plays a key role in various neurobiological theories of consciousness, suggesting that neural activity in thalamo-cortical loops give rise to conscious experience” (Preller et al., 2019, 2745), citing Ward’s (2011) and Tononi and Edelman’s (1998) theories of consciousness that have forefronted these same cortico-thalamic circuits when discussing potential neurobiological bases of consciousness. Thus, parts of this model of psychedelic effects have also been proposed as a potential explanation of phenomenal consciousness and the hard problem of consciousness.

Supporting the CSTC model, LSD increases global thalamic connectivity (Tagliazucchi et al., 2016; Preller et al., 2018a), thalamus-to-cortex connectivity (Müller et al., 2017), and effective thalamic connectivity to both the cortex and the striatum while reducing effective cortico-to-thalamic connectivity (Preller et al., 2019). Increased thalamic connectivity has been associated with the perceptual and other subjective effects of LSD (Müller et al., 2017). Animal models of the effects of psychedelics on pre-attentive sensory gating have provided supporting evidence for the CSTC model (reviewed in Geyer et al., 2001). However, findings of the effects of psychedelic drug administration on thalamic metabolic activity (Hermle et al., 1992; Vollenweider et al., 1997; Gouzoulis-Mayfrank et al., 1999) are mixed. In general, the CSTC model appears be a productive theory for generating hypotheses that better specify the circuitry supporting perception, attention, and executive function through the study of psychedelic drug action on these systems. The CSTC model may thus help to explain how the contents of sensory and interoceptive stimuli come into awareness; however, there appears to be little reason to hope for an answer to the hard problem of consciousness.

Another model of psychedelic effects, the claustro-cortical circuit model, emphasizes the role of the claustrum in mediating psychedelic drug effects. The claustrum is a thin brain structure positioned lateral to the putamen and between the external and extreme capsules, and which is heavily interconnected to a large number of cortical regions (Mathur, 2014). In Francis Crick’s final paper (Crick and Koch, 2005), he and Cristof Koch proposed that the claustrum, due to its widespread cortical connectivity, may provide a multisensory binding function and thus may be a key brain structure for understanding how phenomenal conscious experience may arise from brain function. Here is yet another high-profile claim that a particular brain function (that has also been featured in models of the effects of psychedelics) could explain phenomenal consciousness. Crick and Koch (2005) used the metaphor of the claustrum as a “conductor” and the cortex as an “orchestra” to illustrate how the claustrum might coordinate the activity of functionally and spatially disparate brain regions give rise to phenomenal consciousness. More recent work does not support this binding function for the human claustrum (Bickel and Parvizi, 2019). The potential role of the claustrum in mediating altered states of consciousness was more recently proposed, given the high density of neural receptor targets of classic (i.e., the serotonin 2A receptor) and atypical (e.g., the kappa-opioid receptor, target of salvinorin A) hallucinogens (Nichols, 2016). It has further been proposed that amplification of neuronal avalanches (bursting among neuronal populations that can achieve a supercritical spread of activity) via 5-HT2A activation in the claustrum may be a mechanism of psychedelic action (Nichols et al., 2017). A possible claustrum-cortical model of psychedelic drug action was subsequently supported from empirical observations showing that psilocybin alters connectivity between the claustrum and both the default mode and fronto-parietal attention networks in the human brain (Barrett et al., 2020). Such networks were also identified as central to claustrum involvement in supporting cognitive control under non-drug conditions (Krimmel et al., 2019). This observation is supported in animal findings, which indicate a role for the claustrum in mediating cognitive processes under high cognitive demand or sensory conflict (Atlan et al., 2018; White et al., 2018, 2020). Initial evidence suggests that psychedelics alter the instantiation of cortical cognitive networks through changes in claustrum activity (Barrett et al., 2020). Just as in the 2 previous neural models, and contrary to Crick and Koch’s conjectures, there is no current evidence suggesting a testable hypothesis of the hard problem of consciousness.

Each of these models (REBUS, CSTC, and the claustro-cortical circuit model) may generate testable hypotheses regarding key aspects of subjective experience, including affect, and elements of cognition. There are also other models of psychedelics that relate to claims about consciousness (e.g., Bayne and Carter, 2018; Cofré et al., 2020; LeDoux and Lau, 2020) about which similar conclusions can be drawn. In each case, psychedelics play an important role in testing the parameters of these models and, in some cases, may provide important insights to various “easy problems” of consciousness through their use as a research tool. However, no apparent explanation lies in any of these models for the hard problem of consciousness.

## The Subjective Effects of Psychedelics and the easy Problems of Consciousness

In addition to neuroscientific models of consciousness listed above, there are operationalizations of consciousness that fall within the easy problems of consciousness that focus on carefully characterizing particular aspects of conscious awareness. For example, within phenomenal consciousness, there is a long-standing scientific question on whether consciousness should be considered continuous or discrete. While phenomenal consciousness appears continuous (imagine watching someone walk across your field of vision or hearing a melody), it appears so despite the fact that we only have access to individual moments of time at any given instant. Scientific theories of this kind of conscious awareness generally involve formal models that propose varying degrees and stages of non-conscious processing preceding conscious awareness (Herzog et al., 2020). These theories involving conscious perception are tested in experimental paradigms such as the attentional blink task, which demonstrates that varying the duration of extremely rapid presentation of stimuli can reveal differentiable non-conscious and conscious perception processes (Sergent et al., 2005). Another common task in this context is binocular rivalry, which involves presenting 2 images that compete for perceptual dominance and then determining behavioral or neural correlates associated with perceiving one or the other image (Imamoglu et al., 2012). When psilocybin was administered during the binocular rivalry task, it reduced the rate of binocular rivalry switching (Carter et al., 2007). It will continue to be valuable to test the impact of psychedelics on these and related tasks and in the context of these and other models. Nevertheless, there is little reason to think that states evoked by psychedelic drugs will have a different kind of effect on these models and their related tasks compared with states evoked by any other psychoactive substance. Effects of different psychoactive drugs on these tasks instead will likely differ in terms of degree.

One meaning of the term “consciousness” where psychedelics clearly do have potential to aid scientific investigation is their capacity to produce substantially altered states of consciousness of a particular kind. Altered states of consciousness are defined in the subjective terms of a deviation of aspects of one’s awareness, such as perceived deviations in perception, affect, and cognition from “normal,” or one’s most common waking state of awareness (Tart, 1972; Blackmore, 2013). There is a continuum of alterations from one’s normal state of consciousness, with relatively minor effects induced by caffeine, for instance, to the more intense alterations to perception, affect, and cognition resulting from psychedelic substances (i.e., the effects of LSD differ from those of a cup of tea). Beyond the mere magnitude of the difference from “normal” or “ordinary” states of consciousness, there are questions embedded in this meaning of consciousness regarding the dimensionality of altered states of consciousness (discussed below).

The particular altered states of consciousness elicited by psychedelics have been characterized in several different ways. Psychedelics have purportedly been used for thousands of years in ritual contexts across a number of different cultures, presumably due to their capacity to alter the contents of consciousness in a way deemed meaningful and usually connected to religio-spiritual beliefs (Schultes, 1969). When scientific research began on psychedelics in the mid-20th century, they were initially studied for their potential “psychotomimetic” properties (i.e., mimicking a state of psychosis similar to acute phases of schizophrenia; Osmond, 1957). This pathologizing characterization was disputed after further research demonstrated that psychedelics (taken under supportive conditions) can produce positive experiences with beneficial consequences and limited impairment of reality testing, as opposed to the suffering and negative consequences typically experienced by individuals with psychotic disorders (Johnson and Griffiths, 2017).

The specific altered states of consciousness induced by psychedelics are generally characterized as potentially including a heightened sense of connection, complex imagery, synesthesia, and/or other changes to perception and cognition, as often measured with self-report using the 5-dimensional altered states of consciousness scale (Dittrich et al., 2010; Studerus et al., 2010). Additionally, psychedelics appear to influence one’s subjective sense of self––or the lack thereof––which is commonly referred to as ego dissolution (Nour et al., 2016) or self-transcendent experience (Yaden et al., 2017a). These are changes to one’s awareness of their sense of self or to self-consciousness.

Drawing on scholarship from William James (1902) and others (e.g., Stace, 1960; Hood, 1975), particular altered states of consciousness that can be elicited by psychedelics have also been characterized as “mystical-type” experiences, routinely measured with instruments such as the Mystical Experience Questionnaire 30 (Barrett et al., 2015), which has 4 factors that evaluate (1) feelings of unity and changes to the sense of self, profundity, and that the experience seems extraordinarily real (sometimes referred to as the noetic quality); (2) positive emotions; (3) alterations to the senses of time and space; and (4) ineffability (that the experience is difficult to adequately describe using words). There are undoubtedly a number of more specific cognitive and affective processes at play within these broad factors. Importantly, psychedelics have proven a reliable means to elicit such mystical-type effects (Griffiths et al., 2006, 2008, 2011, 2016; Liechti et al., 2017), and these experiences have been associated with a number of persisting benefits in both healthy and clinical samples (Johnson et al., 2014; Bogenschutz et al., 2015; Ross et al., 2016; Griffiths et al., 2016, 2018; Roseman et al. 2018; Davis et al., 2020). If one is particularly interested in altered states of consciousness of the kind described here (including similar altered states arising from other triggers; see Yaden et al., 2017a), then psychedelics are likely the most important research tools currently available given the relative reliability and magnitude in which they can occasion such effects. As such, psychedelics appear to be powerful tools for investigating some kinds of substantially altered states of consciousness in controlled laboratory settings, and understanding the ramifications of such altered states may be important for understanding the effects of these drugs on mental health.

The kind of altered states of consciousness that psychedelics elicit are presumably influenced by the specific psychedelic substance as well as the apparent dose, expectations, context, bioavailability of the given drug formulation, and many other biopsychosocical factors specific to the psychedelic experience (Studerus et al., 2012; Johnson, Hendricks, Barrett, and Griffiths, 2019; Holze et al., 2021). The nomological network of altered states of consciousness elicited by psychedelics is currently poorly specified, likely due to the unusual nature of these effects and the relative lack of systematic research of such effects in controlled laboratory settings. Psychedelic research provides the possibility of standardizing many features of the dose and the surrounding circumstances under which such altered states of consciousness occur. Notably, the measure of other kinds of mental states, like emotions, was just a few decades ago perceived as too vague and inherently subjective for scientific study (Mlot, 1998); however, there are now robust findings regarding the characterization of emotions (e.g., dimensions of valence and arousal) and their various physiological and behavioral effects. It is possible that psychedelics will allow altered states of consciousness to follow a similar path of scientific inquiry as emotions by providing a reliable means to induce and influence them in controlled settings.

Beyond the measurable changes described above, other changes occur during psychedelic experiences that could be described as even more abstract psychological processes. Many of these processes could broadly be construed as involving changes to one’s self-consciousness, or awareness of aspects of one’s self (e.g., bodily self, perspectival self, volitional self, narrative self, social self; Seth, 2018). Individuals undergoing psychedelic experiences often have experiences and insights pertaining to certain features of their own sense of self-awareness. These experiences may include mental processes related to the sense of self, autobiographical reflection, or perceiving meaningful eidetic imagery. Other insights may include existential concepts such as the potential embodied nature of the mind or how some suffering is optional. Such insights may come about due to the aforementioned subjective experiences that can be occasioned by psychedelics. The creativity literature, for example, discusses similar insights in the concept of “diversifying experiences” (Ritter et al., 2012), which may involve cognitive restructuring of fundamental beliefs after encountering an experience that is far from your “normal” or modal set of everyday experiences. While psychedelics may provide some individuals with some insights into profound-seeming features of their own consciousness, this does not mean that these insights from psychedelic experiences necessarily play a role in identifying scientific ground truth beyond that of introspective insights in general (Johnson, 2020). To be clear, the “insights” that people have under the influence of psychedelics may be false (Letheby, 2019), although the relative frequencies of true and false insights that people tend to have under these conditions remains an open question.

Finally, psychedelics, and the type of altered states they produce, may alter one’s beliefs about the nature of consciousness. Some have proposed that psychedelic experiences may prompt people to move away from materialist views regarding the nature of consciousness, and correlational data suggest this can occur to some degree in some samples (Garcia-Romeu et al., 2015; Yaden et al., 2017a; Griffiths et al., 2019); however, crucially, the magnitude of these changes and the population rates at which these occur are not known. This may be due, at least in part, to the noetic sense associated with psychedelic states, which leads people to feel as though what is intuited or perceived in this state has the quality of feeling real––often somehow “more real” than one’s ordinary state of mind. Such subjective changes may contribute to statements concerning “levels of consciousness,” or abstract sensations of intensity involved with one’s attention and awareness. Feelings of unity and ego dissolution elicited by psychedelics may contribute to alterations in attribution of selfhood and agency (Preller et al., 2018b; Smigielski et al., 2020). Changes to cognitive modules related to mind perception (Gray et al., 2007), which involves the attribution of the capacity for experience and agency to entities/objects, during some altered states of consciousness may also impact one’s beliefs related to what else (other than human beings) has phenomenal consciousness (Yaden et al., 2017b; Nayak and Johnson, 2020). However, a survey of professors of philosophy (Yaden and Anderson, 2021) found no association between psychedelic use and non-materialist views of consciousness, providing evidence that this relationship varies across samples. While further research is required to clarify such effects, it is certainly possible to measure people’s beliefs about the nature of and explanations of consciousness as well as how psychedelic experiences tend to impact those beliefs.

## Conclusion

The scientific study of psychedelics and consciousness, in all of its meanings, is still nascent. While we cannot, at present, see any clear scientific traction resulting from the intersection of psychedelics and the hard problem of consciousness, we are open to the possibility of being proven wrong. We find the relationship between psychedelics and consciousness (in every sense) fascinating, but we also believe that it is important to be clear about which sense of the term consciousness is being used at any given time and to ensure claims regarding explanations of phenomenal consciousness are differentiated from claims about the contents of consciousness (which includes the set of changes to perception, affect, and cognition in what are called altered states of consciousness). We believe this is essential for clear communication among scientists as well as in public science communication (Johnson, 2020). While psychedelics surely impact many phenomena associated with the easy problems of consciousness (though they may not do so uniquely), we believe epistemic humility is called for regarding the potential for psychedelics to illuminate the hard problem of consciousness. Due to the epistemological questions surrounding consciousness, it currently appears unlikely that psychedelics, like other extant scientific tools, could be used to definitively explain the existence of or biological basis of phenomenal consciousness (i.e., solve the “hard problem”); however, psychedelics are proving useful tools for researchers investigating many of the so-called easy problems of consciousness, and it seems likely that their full potential to facilitate scientific advances is only beginning to be tapped.

## Statement of Interest

Dr Barrett is a scientific advisor for WavePaths, LLC, which develops music-based interventions for psychedelic therapies. Dr R. Griffiths is on the board of directors of the Heffter Research Institute. Dr Griffiths has also received grants for research from the Riverstyx Foundation and a crowd-sourced funding campaign organized by Tim Ferriss. Dr M. Johnson is in paid advisory relationships with the following organizations regarding the medical development of psychedelics or related compounds: AWAKN Life Sciences Inc., Beckley Psychedelic Ltd., Entheogen Biomedical Corp., Field Trip Psychedelics, Inc., Mind Medicine, Inc., Otsuka Pharmaceutical Development and Commercialization, Inc., and Silo Pharma, Inc. The other authors have no disclosures to report. Drs A. Garcia-Romeu and S. Nayak serve on a Data and Safety Monitoring Board for Maryland Oncology Hematology. Dr Garcia-Romeu is a scientific advisor for ETHA Natural Botanicals and NeonMind Bioscience.
